# Obituary

**Published:** 1917-03

**Authors:** 


Referring to the obituary notice of Dr. "F. S." Kaynor, a graduate of Geneva Medical College, in Amea, Ia., Dec. 22, at the age of 95 (see Feb. issue), Dr. B. P. Hoyer of Buffalo, his grand-nephew, contributes the following additional notes: The initials should be P. P. and not "F. S." Dr. Kaynor having been named for his uncle-in-law, the late Dr. Peter P. Murphy of Royalton, N. Y., with whom he subsequently studied medicine under the old system of preceptorship. Dr. Kaynor was born in Herkimer Co., N. Y. He was a maternal uncle of the late Frederick F. Hoyer, long a practitioner of Tonawanda, where he died Aug. 16, 1912, aged 91 (see Sept. 1912 issue) but the uncle and nephew were of almost exactly the same age. Dr. Kaynor moved to Royalton, N. Y. when a boy. Uncle and nephew began their medical studies in the same class at Geneva Medical College but, after the first session, Dr. Hoyer engaged in other work for a year or two and graduated at the University of Buffalo in 1849 while Dr. Kaynor continued at Geneva where he must have graduated about 1847 or 1848. Dr. Kaynor practiced for a short time at Newfane and Rapids, N. Y., before moving west. It is impressive that both uncle and nephew, playmates and for part of their medical studies classmates, should both have lived past the age of 90.

